# A windowed carbon nanotube membrane for CO_2_/CH_4_ gas mixture penetration separation: insights from theoretical calculation

**DOI:** 10.1039/d2ra02756a

**Published:** 2022-06-06

**Authors:** Feng Miao, Hao Jiang

**Affiliations:** Key Lab of Information Materials of Sichuan Provincial Universities, Southwest Minzu University Chengdu 610041 China; General Education Department, Sichuan Police College Luzhou 646000 China jianghaojh123@126.com

## Abstract

A new class of species-permselective molecular sieves with functionalized nanowindows has been prepared by modifying the armchair single-walled carbon nanotubes (SWNTs) of a pillared graphene membrane, namely windowed carbon nanotube membrane. The mechanism and characteristics of the windowed carbon nanotube membrane for the selective separation of the CO_2_/CH_4_ gas mixture are comprehensively and deeply studied. Selective gas separation has a great dependence not only on the interaction of the gas adsorbing on the graphene membrane and inside the CNT channel but also with the energy barrier for the gas diffusing through the nanowindow. In all the functional nanowindows investigated, CH_4_ is completely rejected by the N/F-modified nanowindows while maintaining extremely high CO_2_ permeability. The CO_2_ permeance of the nanowindows is as high as 10^9^ GPU. It emerged that these windowed carbon nanotube membranes are efficient species-selective molecular sieves possessing excellent CO_2_/CH_4_ selectivity and brilliant CO_2_ capture capability.

## Abstract 2

Final snapshot of the CO_2_/CH_4_ gas mixture separating through the windowed carbon nanotube membrane.

## Graphical abstract

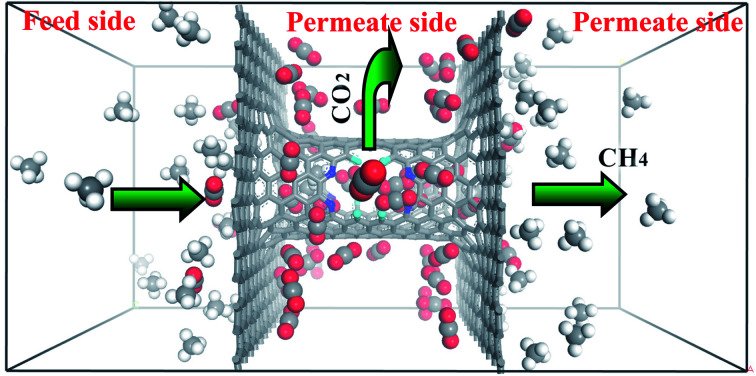

## 1. Introduction

As a mixture containing complex substances, raw natural gas contains several impurities that can increase the gas transmission resistance, which affects its value and prospect in commercial application.^1–3^ To meet the environmental standards and calorific value specifications of renewable energy supplements, the undesirable constituents, such as CO_2_, must be removed before natural gas is delivered to the pipeline network.^4^ To achieve this target, natural gas purification needs to be conducted before its commercial application, which involves the CO_2_/CH_4_ gas mixture separation processes. CO_2_/CH_4_ gas mixture separation is also vital in landfill gas recovery and improving oil recovery.^5,6^ Simultaneously, the captured CO_2_ can be used as a chemical raw material for industrial precursors such as syngas, polycarbonate, and polyurethane. Hence, it is a meaningful work to explore effective materials and methods for CO_2_/CH_4_ gas mixture separation and CO_2_ capture.

Compared with other species purification methods, including supersonic separation,^7^ adsorption,^8^ and cryogenic separation,^9^ membrane separation technology is regarded as superior to its rivals^10–12^ owing to its advantages of low energy consumption, high efficiency, facile operation, and high tunability. Membranes can be used to separate and capture species according to the different characteristics such as molecular diameter, pore size, permeability coefficient, and charge.^13–15^ The graphene-based molecular sieve membranes generally exhibited ultrahigh species flux and selectivity, which far exceed other existing membranes by several orders of magnitude.^16–20^ In this article, graphene-based composite materials are selected as molecular sieve membranes for CO_2_/CH_4_ gas mixture separation and CO_2_ capture.

The permeation and diffusion of the CO_2_/CH_4_ gas mixture through carbon molecular sieve membranes can be impacted dramatically by many factors, including the functionalization of the pore rim and surface.^21–23^ This is because the functionalization of carbonaceous materials can tailor the interaction energy between the molecular sieve and the species, which may facilitate or hinder the diffusion of certain species. For example, Xue *et al.*^24^ found that the selectivity of CO_2_ over N_2_ can be significantly improved by N-functionalized pores because of the enhanced electrostatic interactions when CO_2_ permeates through the functionalized pores. Lu *et al.*^25^ also reported that the permeability of CO_2_ can be dramatically changed by N-substitutional doping, which can change the electroneutrality of the polyphenylene membrane, resulting in an enhancement of the diffusion barrier for CO_2_. Bai *et al.*^18^ investigated the CO_2_/CH_4_ separation performance of N-functionalized nanoporous graphene membrane through theoretical calculations. They found that the configuration of the pore can be changed significantly by the chemical functionalizations, which can achieve high permeability and selectivity for separating the CO_2_/CH_4_ gas mixture. In this article, N/F-functionalized pores are adopted to separate the CO_2_/CH_4_ gas mixture and capture CO_2_ in graphene-based composite materials.

The fast diffusion and separation of species through CNT has been demonstrated in previous studies.^18,25^ To combine the advantages of CNT and graphene membrane, we proposed a new type of carbon molecular sieve—windowed carbon nanotube membrane—for gas transport and separation. Different styles of functionalized nanowindows are designed on the wall of SWNTs, and a multi-scale computational study was implemented to explore the species separation mechanism and performance of the windowed carbon nanotube membrane. Such a system has two advantages: (1) the graphene sheet can provide an efficient adsorption isolating membrane for the gas, which can adsorb species during gas separation and serve as a storage space and transportation channel for species after gas permeation through the functionalized pores, and the CNT can provide a fast-transport channel for the retentate gas after gas mixture separation; (2) the acceptance of different kinds of gas molecules by CNT and nanowindow after gas separation can yield high species permeability and selectivity.

The theme of this paper is to implement a systematic and comprehensive exploration on the permeation and separation characteristics of CO_2_/CH_4_ gas mixtures in a new type of carbon molecular sieve, which are mimicked by windowed carbon nanotube membranes with different window sizes and functional groups using the DFT method and MD simulations. To begin with, the separation mechanism of the CO_2_/CH_4_ gas mixture in the windowed carbon nanotube membrane and the corresponding electronic properties are investigated by performing density functional theory (DFT) calculations. Subsequently, the separation characteristics of the CO_2_/CH_4_ gas mixture under different conditions are elucidated with molecular dynamics (MD) simulation in detail. Finally, the results are summarized at the end of the study.

## 2. Model and computational details

### 2.1. Model

**Fig. 1:**
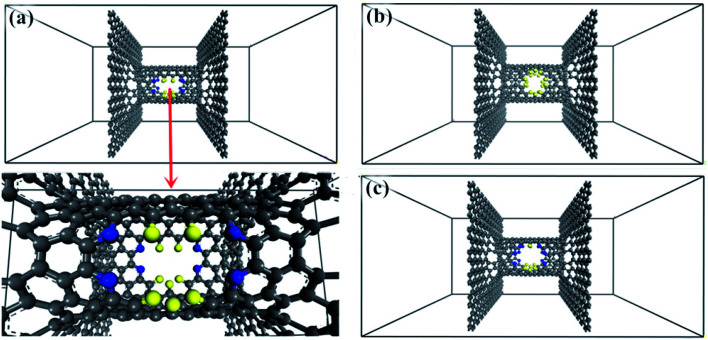
Construction of windowed carbon nanotube membrane model. (a) 4N5F-pore-13, (b) all-F-pore-16, and (c) 6N4F-pore-16 (gray balls represent carbon atom, blue balls represent nitrogen atom, and yellow balls represent fluorine atom).

The windowed carbon nanotube membrane model was envisaged as a perpendicular combination of an armchair (8, 8) windowed SWNT and bilayer graphene sheets, as presented in Fig. 1. The graphene sheets are arranged in the AA mode. The length and diameter of the CNT are set at 2 nm and 1.085 nm, respectively. The CNT decorated with nanowindows works is used as gas diffusion and separation channels, and the graphene sheet is used as a gas adsorption membrane and isolation boundary. Pairs of nanowindows opposite each other are designed on the middle position of the CNT wall as follows: the window is created by removing carbon atoms on pristine CNT and named according to the number of drilled carbon atoms.^26^

### 2.2. DFT calculations

DFT calculations were performed by the DMol^3^ module in the Material Studio software to calculate the adsorption energy of gas molecules on the graphene membrane, the interaction between the gas molecules and modified CNTs, and the energy barrier of gas molecules passing through the nanowindows.^27,28^ Periodic boundary conditions on all the models were taken into account. Generalized gradient approximation (GGA) with the Perdew–Burke–Ernzerhof (PBE) functional was conducted to describe the exchange–correlation interaction.^29–31^ To make dispersion correction for DFT, Grimme's method was adopted.^32^ A double numeric quality with polarization functions (DNP) basis set was employed to expand the electronic wave functions. Self-consistent field (SCF) was employed with a convergence criterion of 10^−6^ a.u.^33,34^ The vacuum thickness for the systems is set to 30 Å to avoid interactions between the adjacent models. All models were continuously optimized until the energy, maximum force, and displacement were less than 1 × 10^−5^ Ha, 0.002 Ha Å^−1^, and 0.005 Å, respectively. The interaction energy (*E*_int_) can be defined as

1 *E*_int_ = *E*_model+gas_ − (*E*_model_ + *E*_gas_)

where *E*_model+gas_, *E*_model_, and *E*_gas_ are the total energies after gas molecules interacting with the model, the energy of the model, and the energy of the isolated gas molecule, respectively.

### 2.3. MD simulations

MD simulations were conducted to elaborate on the CO_2_/CH_4_ separation characteristics of windowed carbon nanotube membrane. The windowed carbon nanotube membrane of area 3 nm × 3 nm × 2 nm divided the simulation box into three chambers (Fig. 1). The simulation box of height 6 nm contained 100 molecules for the CO_2_/CH_4_ gas mixtures, *i.e.*, 50 for CO_2_ molecules and 50 for CH_4_ molecules. Periodic boundary conditions were set in the directions parallel to the graphene sheet to avoid the departure of species outside the simulation box during MD simulation, while reflective wall boundary conditions were applied in the direction perpendicular to the graphene sheet. Each simulation was run for 6 × 10^7^ timesteps with a time step of 1 fs. NVT ensemble was adopted at 298 K controlled by the Nose method. The COMPASS force field in the Forcite module was chosen to express the interatomic interactions.^35^ The electrostatic interactions were calculated by the Ewald method with the accuracy of 0.001 kcal mol^−1^, while the van der Waals interaction was treated with the atom-based option with a cutoff distance of 12.5 Å.^31,36,37^ During MD simulations, the windowed carbon nanotube membrane was modeled as a fully flexible structure. To avert the vertical displacement of the windowed carbon nanotube membrane caused by the collisions with the gas particles, one “central” carbon atom in the graphene membrane is immobilized. In this way, the vibration of other atoms in the windowed carbon nanotube membrane in response to collisions with the gas molecules can be retained by this fixed carbon atom. The simulation data was collected every 5 ps for property analysis. To ensure the accuracy of MD simulations, each simulation was repeated three times under the same settings.

## 3. Results and discussion

**Fig. 2:**
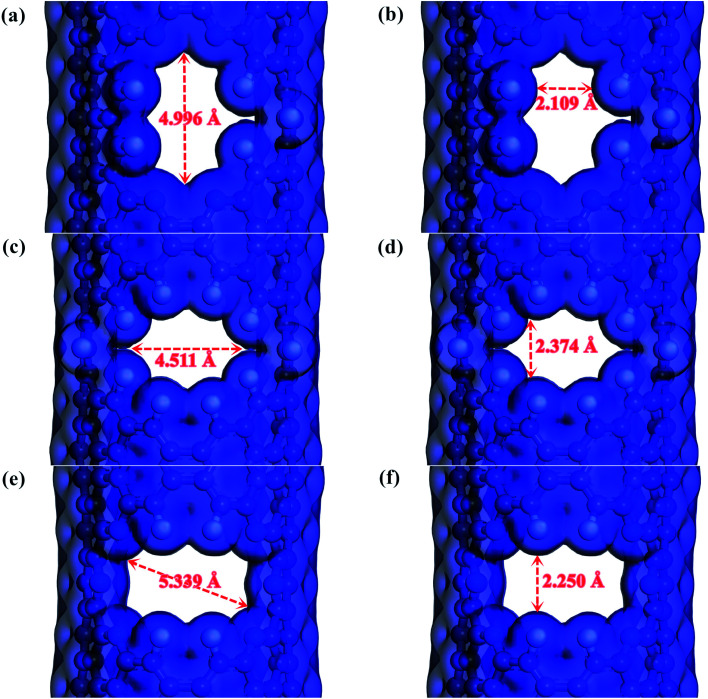
Window electron density isosurface of the windowed carbon nanotube membrane model: (a, b) the maximum and minimum internal distance of the 4N5F-pore-13, respectively; (c, d) the maximum and minimum internal distance of the all-F-pore-16, respectively; (e, f) the maximum and minimum internal distance of the 6N4F-pore-16, respectively. Atom color code: carbon, grey; nitrogen, blue; fluorine, cyan.

Screened by preliminary MD results, three excellent gas separation nanowindows, namely, 4N5F-pore-13, all-F-pore-16, and 6N4F-pore-16, were selected from a series of functionalized pore configurations, as shown in Fig. 1. The nanowindows have a pore size of 3.553, 3.443, and 3.795 Å, respectively, for 4N5F-pore-13, all-F-pore-16, and 6N4F-pore-16, as shown in Fig. 2. These pore sizes are higher than the kinetic diameter of the CO_2_ molecule (CO_2_: 3.3 Å) but lower than the kinetic diameter of the CH_4_ molecule (CH_4_: 3.8 Å). The delicate relationship between the gas molecule's kinetic diameter and the pore size makes windowed carbon nanotube membrane an ideal CH_4_-isolating and CO_2_-capturing apparatus, which can result in the efficient separation of the CO_2_/CH_4_ gas mixture. The atomic distance and bond angle of optimized nanowindows are given in Table 1. The atomic distances of C–C, C–N, and C–F are denoted by l_C–C_, l_C–N_, and l_C–F_, respectively. The bond angles are denoted by ∠C–N–C. Since the substitution of N atoms at the C sites on the nanowindows leads to a distorted pore structure after optimization, l_C–C_ and l_C–N_ are varied in the range of 1.398–1.4822 Å and 1.332–1.359 Å, respectively. ∠C–N–C is varied in the range of 117.937° to 119.344°.

**Table 1:** Summary of structural parameters of various nanowindows

Model	l_C–C_ (Å)	l_C–N_ (Å)	l_C–F_ (Å)	∠C–N–C (°)
4N5F-window-13	1.398–1.465	1.333–1.350	1.337–1.338	117.952–118.988
all-F-window-16	1.400–1.480	None	1.340–1.343	None
6N4F-window-16	1.412–1.482	1.332–1.359	1.332	117.937–119.344

### 3.1. Analysis of interaction energy between the gas molecule and the model

#### 3.1.1. Adsorption energy of the gas molecule on the graphene surface

**Fig. 3:**
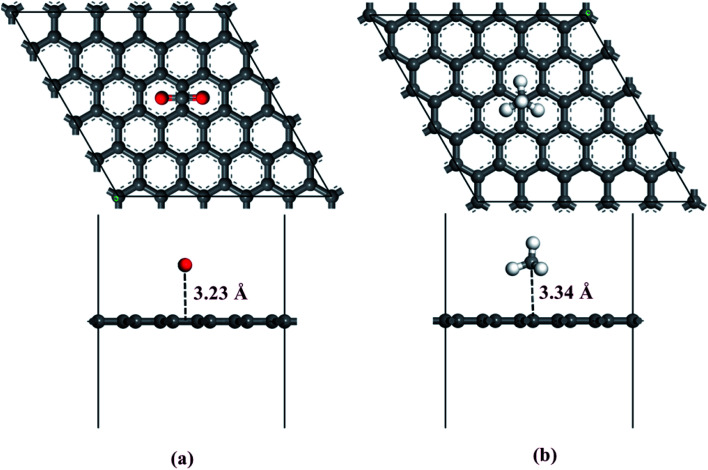
The optimized configurations of CO_2_ and CH_4_ physisorption on the graphene surface. The adsorption of (a) CO_2_ and (b) CH_4_ on the graphene surface. Atom color code: carbon, grey; hydrogen, white; oxygen, red.

The knowledge of the adsorbed states of CO_2_ and CH_4_ on the graphene surface is very important to clarify the mechanisms of CO_2_/CH_4_ gas mixture diffusive separation on graphene materials and to obtain the design guidelines for carbon molecular sieves with CO_2_/CH_4_ gas mixture separation. The adsorbed states for different kinds of gas molecules when the configuration has the lowest energy are presented in Fig. 3. The DFT calculation results demonstrate that the CO_2_ molecule is favorably adsorbed parallel to the graphene membrane, while the CH_4_ molecule prefers to be adsorbed at the top of the carbon atom of the graphene membrane. The optimized configuration of CO_2_ adsorption on the graphene surface is exhibited in Fig. 3(a). The C atom of CO_2_ is located in the middle of the C–C bond of the benzene ring and the O atom of CO_2_ approaches the center of the benzene ring. The adsorption energy of the CO_2_ molecule is about −7.60 kcal mol^−1^. The shortest distance between the carbon atom of CO_2_ and the graphene surface is predicted to be 3.23 Å. The adsorption configuration of the CH_4_ molecule on the graphene plane is also displayed in Fig. 3(b). The C atom of CH_4_ is located on top of the C atom of the graphene membrane and three H atoms of CH_4_ point toward the graphene membrane. The calculated adsorption energy and height of the CH_4_ molecule are −7.42 kcal mol^−1^ and 3.34 Å, respectively. The adsorption of CO_2_ and CH_4_ on the graphene surface are thus categorized into physisorption. The discrepancy of gas adsorption energy results in the selective adsorption of the CO_2_ molecule by the graphene membrane, which can promote the subsequent permeation of the CO_2_ molecule in the CNT channel and inhibit the approach of the CH_4_ molecule around the molecule sieves.

#### 3.1.2. Interaction energy between the gas molecule and the windowed carbon nanotube channel

**Fig. 4:**
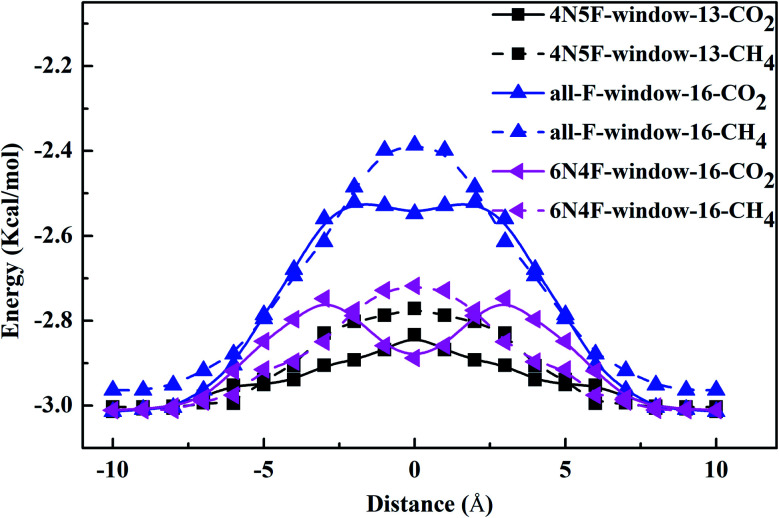
The interaction energy curve between different gas molecules and windowed carbon nanotube channels.

The interaction energy between the gas molecule and CNT also dramatically affects the diffusion order of the species in molecular sieves. The central position of CNT is defined as zero point, and the direction of the coordinate axis is along the CNT. When diffusing in the CNT, the CO_2_ molecule is parallel to the CNT, while three H atoms of the CH_4_ molecule in a plane are parallel to the graphene membrane. Note that the interaction energy between the gas molecules and the windowed carbon nanotube channel shows a decreasing trend with the diffusion of the gas molecule to the middle of the channel, as shown in Fig. 4. The values of the interaction energy between the gas molecules and windowed carbon nanotube channels have a good symmetry corresponding to the nanowindows. The energy minimization of the gas molecule is achieved at or near the middle of CNTs, which is caused by the vdW forces and electrostatic interactions between the gas molecules and defects formed on the wall of the CNTs. The results show that the interaction between the CO_2_ molecule and CNTs is stronger in all the windowed carbon nanotube models. The discrepancy in the interaction energy allows CO_2_ to enter the CNTs before CH_4_.

#### 3.1.3. Interaction energy between the gas molecule and the nanowindow

**Fig. 5:**
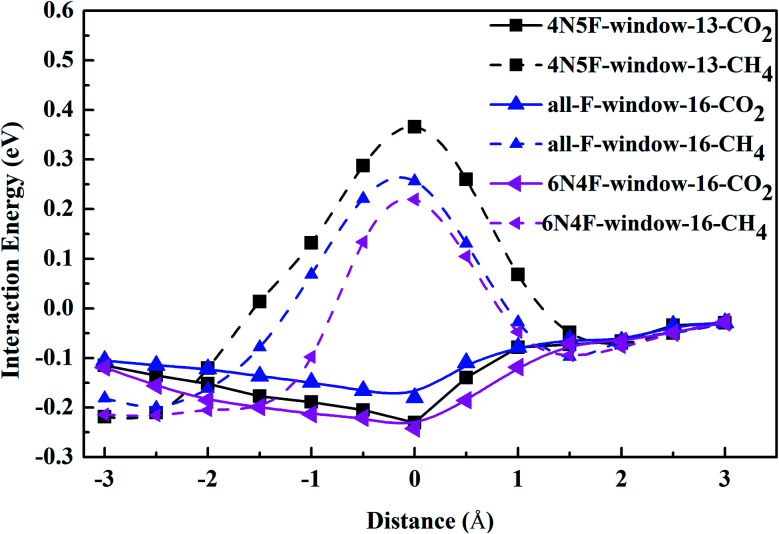
The interaction energy curve between different gas molecules and nanowindows during gas permeation through the nanowindows.

The interaction energy between the gas molecule and the nanowindow was also calculated by eqn (1) provided in Section 2. The line from the carbon atom of the gas molecule to the center of the nanowindow is defined as the *X* axis, which is also known as the adsorption height. Fig. 5 comprehensively shows the resulting interaction energies of CO_2_ and CH_4_ molecules with three different nanowindows. Different values on the *X* axis indicate the distance of the gas molecules from the nanowindow. The negative values on the *X* axis represent gas molecules that move within the nanotube cavity. The result reflects that all the nanowindows show attractive potential energy wells to the CO_2_ molecules, which indicates that it is a spontaneous process for CO_2_ molecules to approach the nanowindow. Therefore, CO_2_ molecules must overcome the attractive potential energy wells to successfully penetrate the nanowindows. Meanwhile, we can monitor that the CO_2_ molecules frequently penetrate forward or return back the 4N5F-pore-13 and 6N4F-pore-16 during CO_2_/CH_4_ gas mixtures separation (see Fig. 7). Accordingly, the highest adsorption energy is achieved for CO_2_ molecules passing through 6N4F-pore-16, whereas the lowest one is achieved for CO_2_ molecules passing through all-F-pore-16. The *E*_int_ for CO_2_ molecules passing through 4N5F-pore-13, all-F-pore-16, and 6N4F-pore-16 are −0.2305, −0.1797, and −0.2422 eV, respectively. Comparing the all-F-pore-16, the *E*_int_ for 4N5F-pore-13 and 6N4F-pore-16 significantly increases about 28% and 35%, respectively. In contrast, the *E*_int_ dramatically increases when the carbon atom of the CH_4_ molecules is close to the center of the pore, which means that CH_4_ molecules must overcome the repulsive potential energy barrier to penetrate the nanowindows. The specifically high attractive potential energy between the CH_4_ molecules and nanowindows can obviously increase the CO_2_ molecules crossing the probability through the single-atom-thick nanowindows so as to obtain excellent CO_2_ selectivity.

**Fig. 6:**
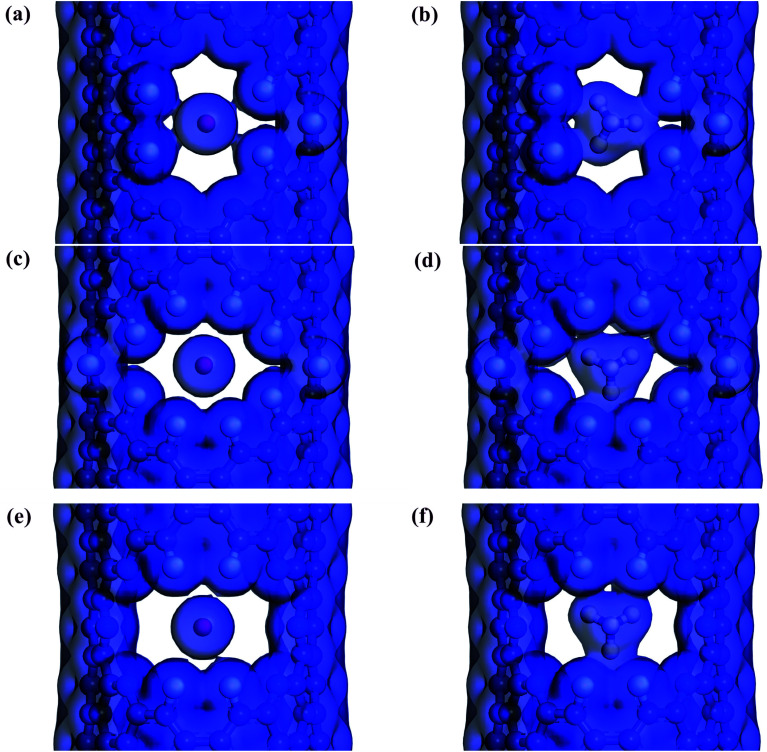
Electron density isosurface for different kinds of gases penetrating through the nanowindows of (a) and (b) 4N5F-pore-13, (c) and (d) all-F-pore-16, and (e) and (f) 6N4F-pore-16, respectively (isovalue of 0.02 e Å^−3^). Atom color code: carbon, grey; nitrogen, blue; fluorine, cyan.

The electron density isosurfaces of the CO_2_ and CH_4_ molecules interacting with different nanowindows are also measured to elucidate the origin of the energy difference during gas penetration through the windows in more detail. As shown in Fig. 6, CO_2_ molecules show no overlap with all three nanowindows, whereas CH_4_ molecules show obvious overlap with all three nanowindows significantly, resulting in a large energy difference when different kinds of gas molecules pass through the functionalized nanowindows. The linear interpenetration of CO_2_ passing the nanowindows is observed in all windowed carbon molecular sieves, while CH_4_ is completely rejected by all the nanowindows. Due to its smaller size, CO_2_ diffusion is favored energetically compared to CH_4_ (Fig. 5). Moreover, CO_2_ passes through the nanowindows by orienting its molecular axis along the window centers, as shown in Fig. 6. Considering the permeation mode of linear molecules in CNTs, CO_2_ molecules typically rotate in the direction of the CNT channel and then perpendicularly through the nanowindow, which is favored in the entropic selectivity. In these circumstances, the CH_4_ molecule is completely blocked out of the nanowindow.

To sum up, according to the detailed investigation and comparison of the interaction energy between the gas molecules and the windowed carbon nanotube membranes, we can find that the preferential adsorption and permeation of CO_2_ contribute partially to the gas mixture separation, while the interaction energy difference in the process of the penetrating nanowindow is the most important determinant in the separation process. Based on the energy barrier information, MD simulation was then performed to study the gas separation process for various molecular sieve models.

### 3.2. The CO_2_/CH_4_ separation performance

**Fig. 7:**
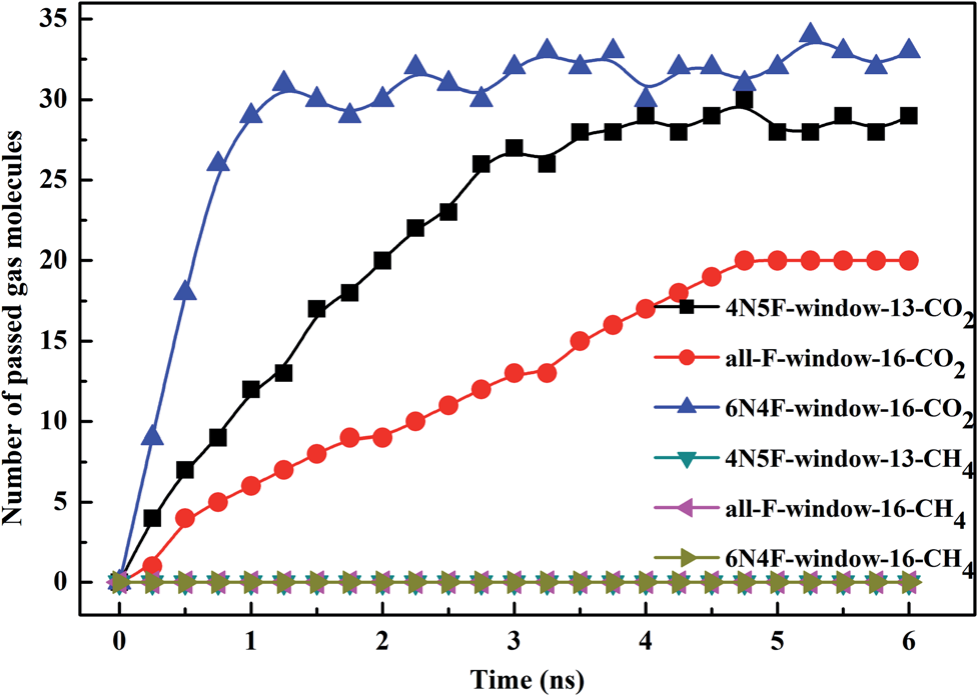
The number of diffusing species in different windowed carbon nanotube membranes as a function of the MD simulation time.

**Fig. 8:**
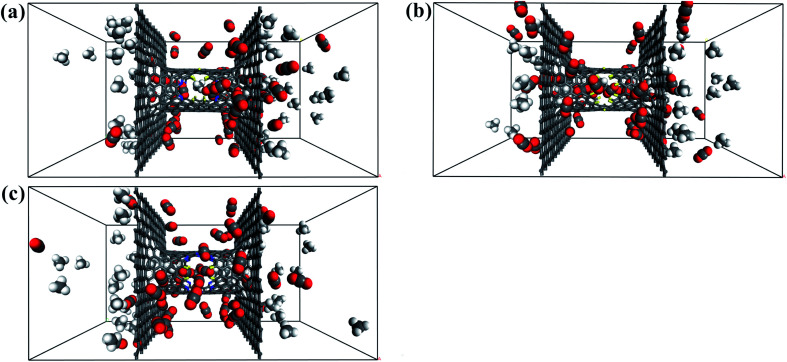
Final snapshots of the CO_2_/CH_4_ gas mixture separating through the windowed carbon nanotube membranes (a) 4N5F-pore-13, (b) all-F-pore-16, and (c) 6N4F-pore-16.

The filtered molecular numbers of the gas through different functionalized nanowindows as a function of time is shown in Fig. 7 and the final snapshots of the CO_2_/CH_4_ gas mixture separation through the nanowindows are presented in Fig. 8. Several interesting phenomena can be concluded: (1) among the various N/F modified nanowindows investigated, only CO_2_ diffusion is observed; CH_4_ is completely rejected by the nanowindows. (2) For all time-dependent CO_2_ penetration curves (Fig. 7), the uphill straight line reflects the same penetration trend of CO_2_ in the initial time region of the pre-equilibrium stage. (3) The permeability of CO_2_ increases significantly with the increase in the effective pore size. The maximum permeability of CO_2_ can be observed with 6N4F-pore-16. (4) The fluctuation of the CO_2_ permeation curve also increases with the expansion of the effective pore size, which is due to the increased possibility of the crossing-back motions of the gas molecules from the feed side to the permeate side. The MD results qualitatively agree well with the speculation provided by DFT calculations. Gas separation through the windowed carbon nanotube membranes involves adsorption and diffusion, and the selectivity is mainly dictated by the interaction energy between the gas molecules and nanowindows. Also, the aggregation of the diffusing particles around the graphene surface region was observed in this study. The permeated CO_2_ molecules spend almost all of their time next to the graphene surface, where they exhibit pressure-independent surface diffusion.

Gas permeance was employed as another criterion to estimate the performance of the windowed carbon nanotube membrane, which was calculated using eqn (2)2
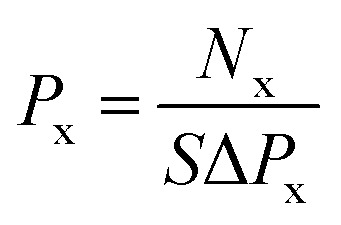
where *P*_x_ represents the permeance of gas x, *N*_x_ represents the permeate rate of the gas x, and *S* represents the area of the windowed carbon molecular sieve. Δ*P*_x_ is set by default to 1 × 10^5^ Pa.^38^ The permeance of the species with different nanowindows is listed in Table 2. Compared to previous studies on porous graphene or windowed carbon nanotube gas separation systems, our gas permeability is increased by 10^4^–10^7^ orders of magnitude.^18,22,39,40^ In addition, the separated gas flows to different permeate sides to avoid mixing with the gas in the original feed side, ensuring that the gas mixture can be separated continuously.

**Table 2:** The permeance of species with different nanowindows

Molecules	4N5F-window-13	all-F-window-16	6N4F-window-16
CO_2_	1.50 × 10^9^ GPU	9.31 × 10^8^ GPU	1.71 × 10^9^ GPU
CH_4_	0	0	0

### 3.3. MD simulation of the CO_2_/CH_4_ separation mechanism of the windowed carbon nanotube membrane

**Fig. 9:**
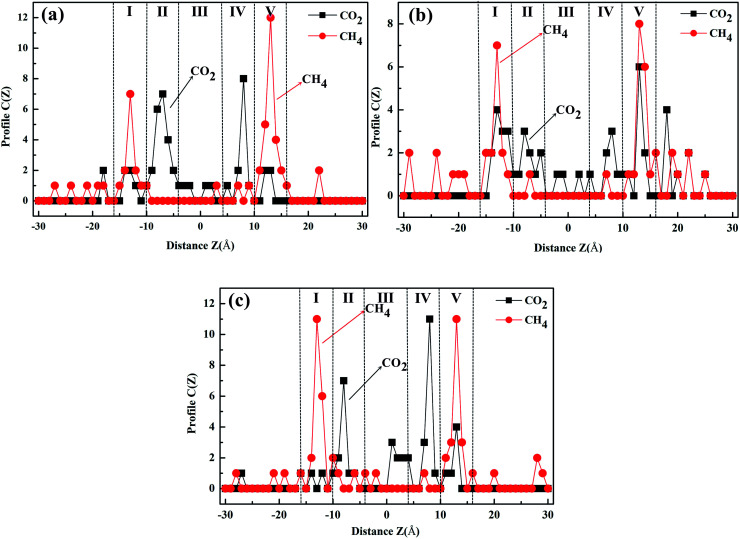
The final number distribution curves of gas molecules along the direction perpendicular to the graphene sheet for the windowed carbon nanotube membrane with different nanowindows. (a) 4N5F-pore-13, (b) all-F-pore-16, and (c) 6N4F-pore-16.

To test and verify the CO_2_/CH_4_ separation mechanism of the windowed carbon nanotube membrane obtained by DFT calculation, the adsorption regions of gas molecules in the different models were studied, and the final number distribution of the gas molecules along the direction perpendicular to the graphene sheet is plotted in Fig. 9. According to the results of gas separation, five gas adsorption regions are formed in the windowed carbon nanotube membrane (region I to region V). For CO_2_ molecules with higher adsorption intensity, apart from being captured by CNTs, several molecules are adsorbed on the graphene surface (the graphene membrane is located at *z* = −10 and 10 Å). These CO_2_ molecules firstly reach region I and then migrate to the vicinity of the functionalized carbon nanotube mouth or are directly adsorbed into the functionalized carbon nanotube before they cross the nanowindows. Meanwhile, region III accumulated by the blockage of CO_2_ molecules near the nanowindows of the windowed carbon nanotube membrane also plays an important role in CO_2_ diffusion characteristics. These CO_2_ molecules have a good barrier effect on the penetration of the CH_4_ molecule. Nearly all the permeated CO_2_ molecules are adsorbed in region II and region IV. The penetration of CO_2_ molecules through the nanowindow leads to a large number of CH_4_ molecules adsorbing in region I and region V, and there is no CH_4_ molecule crossing event that occurs in the nanowindow, thus significantly improving the CO_2_/CH_4_ selectivity. According to the study on the adsorption mechanism, the windowed carbon nanotube membranes show preferential adsorption toward CO_2_, which is mainly attributed to vdW force and electrostatic interaction, which gives CO_2_ molecules more chances to approach the nanowindows. The competitive adsorption mechanisms are beneficial for CO_2_ permeation. It can also be seen from the separation results in Fig. 9(a) and (c) that regions II–IV are basically CO_2_ molecules, and the CH_4_ molecules in them only represent that they happen to be in the CNT channels. After gas separation, the retentate gas in permeate side (10–30 Å regions) are basically CH_4_ molecules. In terms of the gas separation effect, 4N5F-pore-13 and 6N4F-pore-16 are ideal.

### 3.4. Effect of the pressure difference on the CO_2_ permeability

**Fig. 10:**
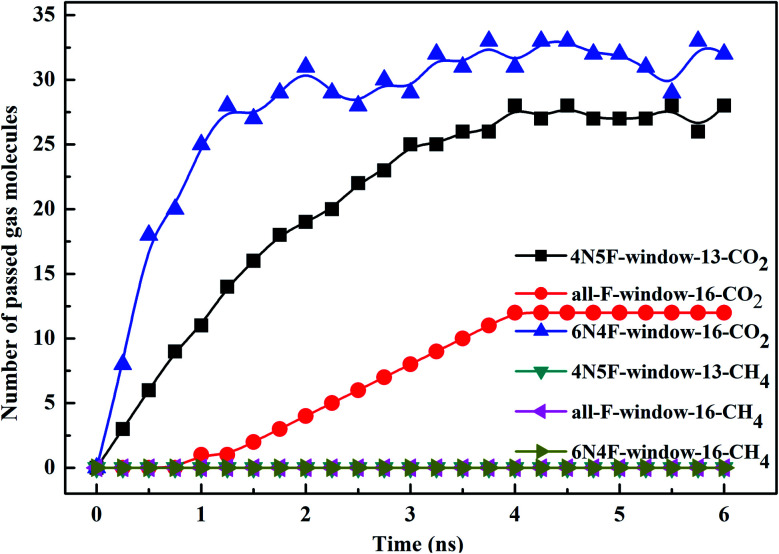
The number of diffusing species in different windowed carbon nanotube membranes obtained without pressure difference.

**Fig. 11:**
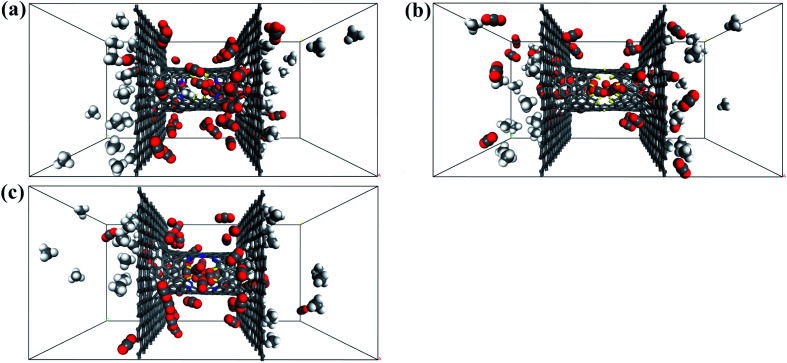
Final snapshot of the CO_2_/CH_4_ gas mixture separating through the windowed carbon nanotube membranes. (a) 4N5F-pore-13, (b) all-F-pore-16, and (c) 6N4F-pore-16.

In this section, the effect of pressure difference on CO_2_/CH_4_ permeation and separation in the windowed carbon nanotube membrane is investigated by MD simulations. For comparison, we calculated the number and permeance of species crossing the nanowindows when equimolar CO_2_/CH_4_ gas mixture was placed on both sides of the windowed carbon nanotube membrane, as displayed in Fig. 10 and Table 3, respectively. The striking selective permeation of CO_2_ passing through the nanowindows is observed in the simulations. This result means that the high surface affinity of the model and the small kinetic diameter of the CO_2_ molecule are able to ensure the selective penetration of CO_2_, regardless of the starting configuration of the gas mixture. The final snapshots of the CO_2_/CH_4_ gas mixture separating through the nanowindows are presented in Fig. 11. However, the results reported in Table 3 show that the permeability of CO_2_ through the nanowindows decreased. This is because when the pressure difference on both sides of the model disappears, the fluidity of the species is weakened, inhibiting the diffusion and permeation of the gas. Moreover, the smaller the pore size of the nanowindow, the more obvious the downward trend of permeability. This is because the smaller the pore size, the smaller the adsorption force acting on the CO_2_ molecules. The all-F-pore-16 has the least affinity for CO_2_ molecules, which leads to the most significant reduction in the number of gas molecules that permeate when the molecular motion slows down. This proves once again the important role of nanowindows in gas separation, especially for models with small-sized nanowindows.

**Table 3:** The permeance of species with different nanowindows

Molecules	4N5F-window-13	all-F-window-16	6N4F-window-16
CO_2_	1.40 × 10^9^ GPU	6.21 × 10^8^ GPU	1.66 × 10^9^ GPU
CH_4_	0	0	0

## 4. Conclusion

In this article, a comprehensive exploration of the CO_2_/CH_4_ gas mixture separation mechanism and the property of windowed carbon nanotube membrane are provided. All the simulation results manifest that the windowed carbon nanotube membrane with N/F atoms functionalized on the pore rim can act as a filtration membrane for the separation or enrichment of CO_2_/CH_4_ gas mixtures with remarkably high CO_2_ permeability and selectivity. The differences in the interaction between the gas molecules and the windowed carbon nanotube membrane, especially the strong repulsive interaction between CH_4_ molecules and nanowindows, resulted in 100% CO_2_/CH_4_ selectivity. It can be deduced that 6N4F-pore-16 has the best permeability on the diffusion of CO_2_ molecules in all the simulated windowed carbon nanotube membrane models. Detailed analysis of the simulated systems reveals that the difference in the interaction potential energy with graphene sheet and CNT, and the electron density overlap of the nanowindow facilitate CO_2_/CH_4_ gas mixture separation through the windowed carbon nanotube membrane.

## Conflicts of interest

There are no conflicts to declare.

## Supplementary Material
